# Two Types of Etiological Mutation in the Limb-Specific Enhancer of *Shh*

**DOI:** 10.1534/g3.117.044669

**Published:** 2017-07-14

**Authors:** Takanori Amano, Tomoko Sagai, Ryohei Seki, Toshihiko Shiroishi

**Affiliations:** Mammalian Genetics Laboratory, Genetic Strains Research Center, National Institute of Genetics, Mishima, Shizuoka 411-8540, Japan

**Keywords:** enhancer, gene regulation, preaxial polydactyly, Sonic hedgehog

## Abstract

An enhancer named MFCS1 regulates Sonic hedgehog (*Shh*) expression in the posterior mesenchyme of limb buds. Several mutations in MFCS1 induce ectopic *Shh* expression in the anterior limb bud, and these result in preaxial polydactyly (PPD). However, the molecular basis of ectopic *Shh* expression remains elusive, although some mutations are known to disrupt the negative regulation of *Shh* expression in the anterior limb bud. Here, we analyzed the molecular mechanism of ectopic *Shh* expression in PPD including in a mouse mutation—hemimelic extra toes (*Hx*)—and in other MFCS1 mutations in different species. First, we generated transgenic mouse lines with a *LacZ* reporter cassette flanked with tandem repeats of 40 bp MFCS1 fragments harboring a mutation. The transgenic mouse line with the *Hx*-type fragment showed reporter expression exclusively in the anterior, but not in the posterior margins of limb buds. In contrast, no specific *LacZ* expression was observed in lines carrying the MFCS1 fragment with other mutations. Yeast one-hybrid assays revealed that the msh-like homeodomain protein, MSX1, bound specifically to the *Hx* sequence of MFCS1. Thus, PPD caused by mutations in MFCS1 has two major types of molecular etiology: loss of a *cis*-motif for negative regulation of *Shh*, and acquisition of a new *cis*-motif binding to a preexisting transcription factor, as represented by the *Hx* mutation.

Sonic hedgehog (*Shh*) encodes a signaling protein that plays indispensable roles during development. In the mouse limb bud, *Shh* is expressed in a group of posterior mesenchymal cells, known as the zone of polarizing activity (ZPA). A noncoding sequence named Mammal–Fish Conserved Sequence 1 (MFCS1; also known as ZRS) is located in a region 860 kb from the *Shh* coding sequence (Lettice *et al.* 2003; Sagai *et al.* 2004). A reporter transgene assay revealed that a 1.7 kb MFCS1 sequence contains limb-specific *Shh* enhancer activity (Lettice *et al.* 2003). Moreover, elimination of MFCS1 caused a specific loss of *Shh* expression in the limb bud (Sagai *et al.* 2005). Thus, MFCS1 is necessary and sufficient for the activation of *Shh* in limb buds. The ZPA-specific expression of *Shh* is regulated by transcriptional activators that directly bind to MFCS1, such as 5′HOXD and HAND2 (Capellini *et al.* 2006; Galli *et al.* 2010). On the other hand, *Shh* is normally repressed in the anterior limb bud, and the impairment of anterior repression causes ectopic expression of *Shh*.

Loss-of-function mutations of several transcription factors (TFs), such as GLI3, ALX4, and GATA6, cause anterior expression of *Shh* and preaxial polydactyly (PPD) (Chan *et al.* 1995; Masuya *et al.* 1995; Qu *et al.* 1998; Takahashi *et al.* 1998; Kozhemyakina *et al.* 2014). Strong’s luxoid (*Lst*), which is a spontaneous mouse mutation, causes loss of DNA-binding activity of Aristaless-like 4 (*Alx4*), and thereby disrupts repression of *Shh* in the anterior limb bud (Takahashi *et al.* 1998). Whether ALX4 directly or indirectly regulates *Shh* via binding to MFCS1 is still not known. A chromatin immunoprecipitation assay revealed that GATA6 directly binds to MFCS1 to suppress the anterior expression of *Shh* in the normal limb bud (Kozhemyakina *et al.* 2014). Thus, loss of repressor function in the anterior limb bud is likely to be a major *trans*-acting mechanism underlying PPD.

A single nucleotide substitution in MFCS1 of human, cat, mouse, and chicken cause PPD (Lettice *et al.* 2003, 2008; Dorshorst *et al.* 2010; Albuisson *et al.* 2011; VanderMeer and Ahituv 2011; Anderson *et al.* 2012). In the mouse, a spontaneous mouse mutation *Hx*, and the N-ethyl-N-nitrosourea (ENU)-induced mouse mutations *M100081*, *M101116*, and *DZ*, have a single nucleotide substitution at different sites in MFCS1, and they all show a typical PPD phenotype with ectopic *Shh* expression in the anterior limb bud (Masuya *et al.* 2007; Zhao *et al.* 2009). Considering loss-of-function mutations of *Alx4* and *Gata6*, a simple explanation of the molecular mechanism underlying PPD is that a mutation in MFCS1 could abolish binding of a transcriptional repressor to MFCS1. To date, >20 MFCS1 mutations in different vertebrates exhibit PPD, and they mostly have the same outcome, in that *Shh* is expressed ectopically in the anterior limb bud. Whether the molecular etiology of each mutation differs from one another is still not clear.

Here, we showed that there are two types of molecular etiology in the MFCS1 mutations that exhibit PPD. One type upregulates *Shh* expression in the anterior limb buds through the loss of binding of a potential repressor, whereas the other type activates ectopic *Shh* expression via new acquisition of a *cis*-motif that binds to a preexisting TF. Furthermore, this study indicated that the *Hx* mutation is an example of the latter type, and it acquired a new motif that binds to a homeodomain protein. Finally, we found that MSX1 is a candidate for this TF.

## Materials and Methods

### Production of transgenic mice and detection of reporter expression

**Figure 1:**
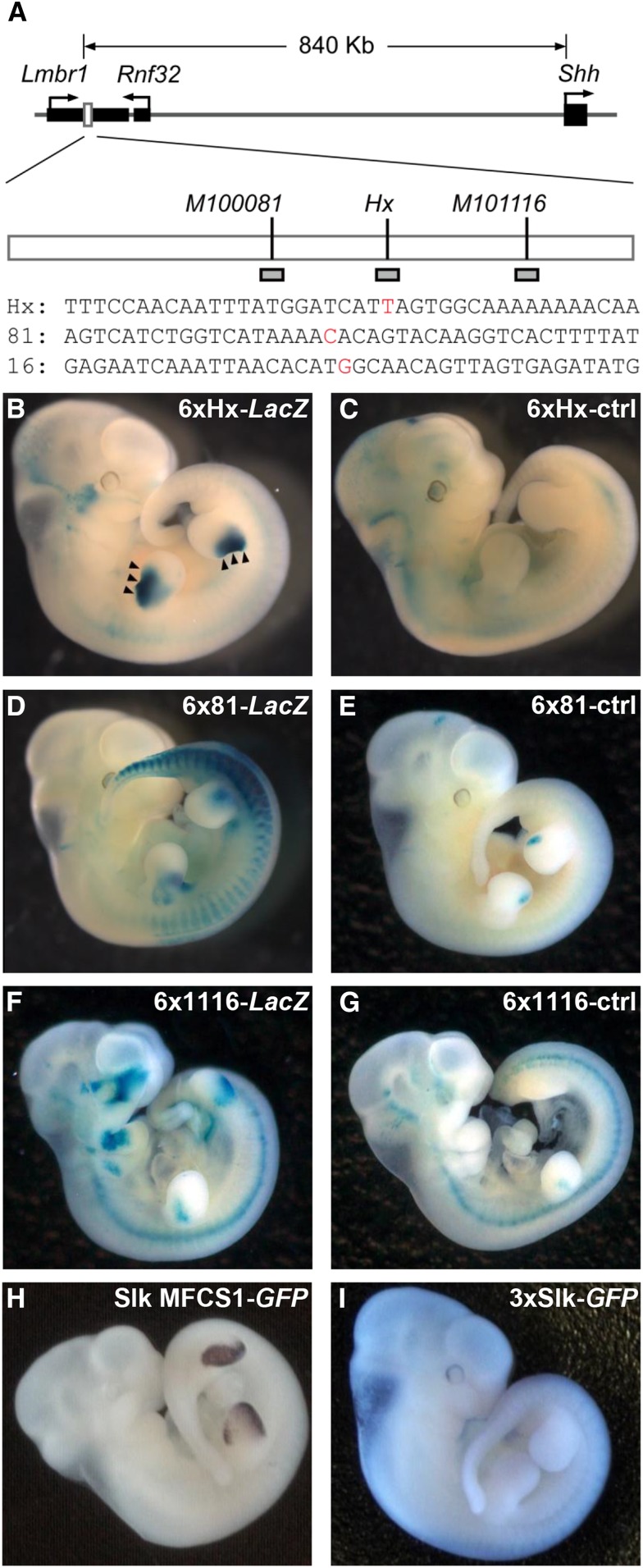
A short *Hx* fragment was sufficient to induce *LacZ* reporter expression in the anterior limb bud. (A) Schematic diagram of the genomic region around the *Shh* locus on mouse chromosome 5. MFCS1 (open box) is located in the intron of *Lmbr1*. Positions of three mouse PPD mutations and short fragments used in the transgenic reporter assay (shaded boxes) are represented. Red letters in sequences indicate nucleotides at the *Hx*, *M100081* (81) and *M101116* (1116) mutation sites. The images of embryos show *LacZ* reporter expression driven by tandem repeats of the mutant sequences (B) 6xHx-*LacZ*, (D) 6x81-*LacZ*, and (F) 6x1116-*LacZ*, and the corresponding WT genome sequences (C) 6xHx-ctrl, (E) 6x81-ctrl, and (G) 6x1116-ctrl. Also shown is GFP expression at E11.5 in the Silkie MFCS1-*Gfp* transgenic mouse (H) and in the transgenic mouse embryo containing three tandem repeats of the *Slk* fragment (I).

**Figure 2:**
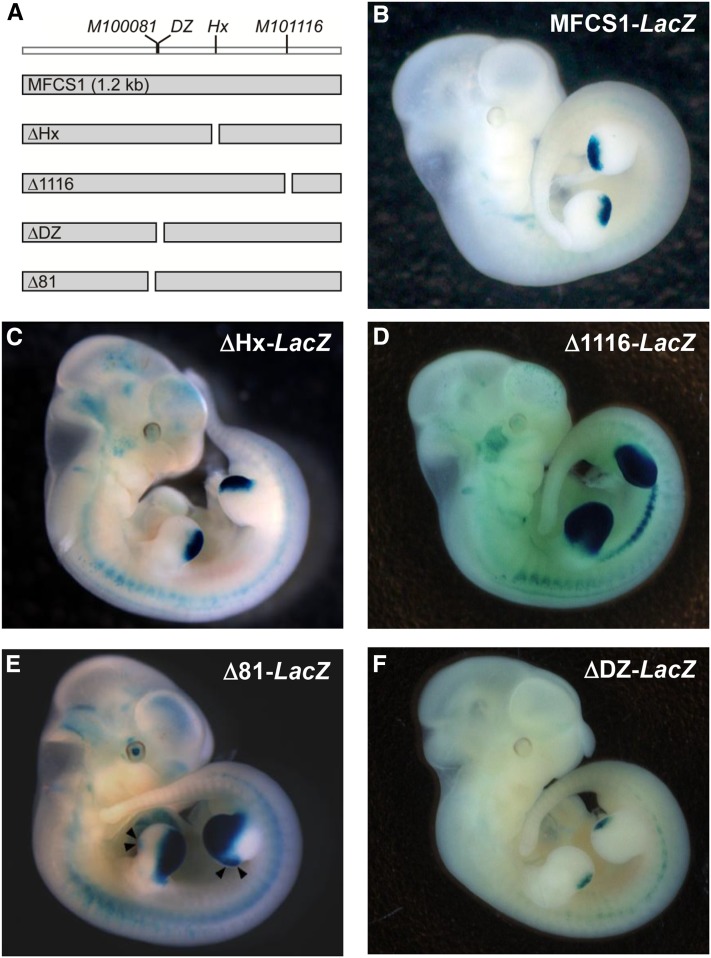
Effect of single nucleotide deletions on *LacZ* reporter expression in MFCS1-*LacZ* transgenic mice. (A) Schematic diagram of single nucleotide deletions in MFCS1. *LacZ* expression was examined in E11.5 transgenic embryos with the 1.2 kb MFCS1 enhancer (B) MFCS1-*LacZ*, and MFCS1 containing a single-base deletion at the *Hx* mutation site (C) ΔHx-*LacZ*, at the *M101116* site (D) Δ1116-*LacZ*, at the *M100081* site (E) Δ81-*LacZ*, and at the *DZ* site (F) ΔDZ-*LacZ*. Arrowheads indicate an anterior expression domain of *LacZ* in the limb bud.

Tandem short fragments (Figure 1 and Supplemental Material, Table S1 in File S2) and the whole MFCS1 (Figure 2) were PCR amplified, and cloned into the HSF51 vector, which contains the *Hsp68* promoter and a *LacZ* reporter cassette. Transgenic mice were generated by pronuclear microinjection of *LacZ* transgenes into zygotes derived from (C57BL/6 × DBA/1) F1 intercrosses as described (Sagai *et al.* 2009). X-gal staining and *in situ* hybridization were performed as reported (Tsukiji *et al.* 2014). Immunostaining of GFP was performed as reported (Amano *et al.* 2009), with minor modifications.

### Yeast one-hybrid assay

Yeast one-hybrid screening was carried out using the Matchmaker Gold Yeast One-Hybrid Library Screening System (Clontech), according to the manufacturer’s protocol. Three tandem repeats of the 20 bp fragment containing the *Hx* mutation was cloned into pAbAi reporter vector, and then integrated into the Y1HGold yeast genome. Total RNA was isolated from the limb buds of E11.5 mouse embryos using RNeasy mini column kits (Qiagen). The cDNA library was generated according to Clontech’s SMART technology. For the first screening, the cDNA and linearized pGADT7 vector are cotransformed into the Y1HGold [*Hx*-pAbAi], and transformants were plated in SD medium-Leu formula with 200 ng/ml Aureobasidin A (AbA). We screened 0.18 million transformants and obtained 45 clones. After DNA sequencing, 15 out of 45 clones encoded part of a protein-coding gene. For the second screening, 20 bp of the wild-type (WT) sequence corresponding to the *Hx* fragment was used to generate the Y1HGold [ctrl-pAbAi], and the 15 clones were transformed individually. Clones that did not survive were considered to be true positives.

The pGAD-*Msx1*-AD clone and a control empty vector, pGADT7-AD, were used to verify the DNA–protein interaction. These clones were transformed into both Y1HGold [*Hx*-pAbAi] and Y1HGold [ctrl-pAbAi] and spotted onto agar plates with SD medium-Leu formula with or without AbA. The pGADT7-Rec-*p53* vector and Y1HGold [p53-pAbAi] yeast strains were used as positive controls.

### Electrophoretic mobility shift assay

Complementary pairs of oligonucleotides were annealed and end-labeled with ^32^P-ATP or digoxigenin. The following oligonucleotide sequences were used for making probes: 5′-TTATGGATCATTAGTGGCAA-3′ for the *Hx* probe; 5′-TTATGGATCATCAGTGGCAA-3′ for the WT probe; 5′-ATTCGATCGGGGCGGGGCGAGC-3′ for the SP1 probe. The probes were incubated with 10 μg of nuclear extract from whole bodies or limb buds of E11.5 mouse embryos. A poly(dI–dC) competitor was added to prevent proteins from binding nonspecifically.

### Cell culture and luciferase assay

Full-length or tandem copies of MFCS1 were inserted upstream of a minimal promoter and firefly luciferase in the plasmid pGL4.23 (Promega). The plasmid pGL4.74 ubiquitously expressing *Renilla* luciferase was used as a control. Protein-coding sequences of interest were inserted into pcDNA3.1 (Life Technologies), which is an expression vector in mammalian cells. NIH3T3 and Caco-2 cells were maintained in 10% fetal bovine serum/Dulbecco’s modified Eagle’s medium containing 100 μg/ml penicillin–streptomycin. Cells at 70–90% confluency were transfected with firefly and *Renilla* luciferase reporter plasmids, and with expression plasmids using Lipofectamine 3000 (Invitrogen). Luciferase activity in the cell lysates was measured using a Dual-Luciferase Reporter Assay System (Promega) according to the manufacturer’s protocol.

### Data availability

All plasmids and primer sequences used in this study are available upon request. The authors state that all data necessary for confirming the conclusions presented in the article are represented fully within the article.

## Results and Discussion

### A short Hx-type MFCS1 fragment is sufficient to evoke anterior ectopic expression of Shh

A transgenic MFCS1 fragment with a single nucleotide substitution that was observed in PPD animals induced anterior ectopic expression of a *LacZ* reporter in the mouse developing limb (VanderMeer and Ahituv 2011; Anderson *et al.* 2012). However, it is hard to determine whether gain or loss of TF-binding occurs at MFCS1 mutation sites when the entire MFCS1 enhancer is used in the mouse transgenic reporter assay. To examine whether a short genomic fragment covering a single PPD mutation would be solely sufficient to activate gene expression, we generated transgenic mice with the *LacZ* reporter flanked by tandem repeats of 40 bp sequences harboring three mouse mutations (Figure 1A and Table S1 in File S2). If a short fragment with a PPD mutation induces an anterior expression of *LacZ* in the limb bud, we infer that gain of TF-binding occurred in the PPD animals.

In transgenic mouse embryos at E11.5, a 6× tandem repeat of a 40 bp fragment containing the *Hx* mutation induced *LacZ* expression exclusively in the anterior limb bud (Figure 1B). Notably, these embryos had no expression in the posterior limb bud where *Shh* is normally expressed. The control WT sequence at the *Hx* mutation did not induce *LacZ* expression in the limb bud (Figure 1C), suggesting that this anterior expression depends on the *Hx* mutation. In contrast to *Hx*, a 6× repeat of a 40 bp fragment containing the *M100081* mutation induced neither anterior nor posterior expression of *LacZ* (Figure 1D). On the other hand, in two out of seven transgenic embryos, a 40 bp fragment with the WT sequence at the *M100081* mutation induced *LacZ* expression in the ZPA, but not in the anterior mesenchyme (Figure 1E). This result supports unknown motif(s) in the 40 bp WT fragment around the *M100081* mutation taking part in normal *Shh* regulation in the ZPA. A 6× tandem repeat of a 40 bp fragment around the *M101116* mutation induced *LacZ* in an anterior middle part of the limb bud, irrespective of the presence or absence of the mutation (Figure 1, F and G). The expression domain was not restricted to the anterior edge of limb buds, and was different from that induced by the 6× *Hx* fragment. The *Hx* mutation results in an ATTA sequence (Figure S1 in File S1), which is known to be a core homeodomain-binding motif (Catron *et al.* 1993). Of known PPD mutations in MFCS1, the chicken Silkie mutation (*Slk*) also generates this motif (Dorshorst *et al.* 2010; Maas *et al.* 2011; Johnson *et al.* 2014). The entire *Slk* MFCS1 sequence upregulated reporter expression in both the anterior and posterior margins of the transgenic mouse limb buds (Figure 1H), whereas tandem repeats of a 40 bp fragment containing the *Slk* mutation did not drive such reporter expression (Figure 1I). This result suggests that the mechanism by which the anterior ectopic *Shh* expression is elicited in *Slk* is different from that in *Hx*, although both mutations create an ATTA motif.

Single nucleotide substitutions in the human PPD mutations mostly occur at nucleotide positions that are highly conserved between the human and mouse genomes (Lettice *et al.* 2003). We examined the regulatory activity of five human mutations on short mouse sequence backbones by mouse transgenic assays (Table S1 in File S2). The result showed no specific expression of the *LacZ* reporter in the transgenic mouse limb buds (Figure S2, A–E in File S1 and Table S2 in File S2).

In summary, out of all mutations in the human, mouse, and chicken genomes that we examined, the *Hx* short fragment clearly showed gain of a new activity to induce anterior *Shh* expression. It is noted that we cannot rule out the possibility that other MFCS1 mutations also gained a new regulatory activity, because the fragments used for the reporter transgenic assay were only 40 bp and may have lost the context of limb bud–specific regulation. Previous studies, in fact, reported that unknown nuclear factors can bind to MFCS1 with specific mutations (Farooq *et al.* 2010; Fuxman Bass *et al.* 2015), although it is still unclear whether these nuclear factors act as transcriptional activators *in vivo*.

### A specific single-base substitution at the Hx mutation site is indispensable for the anterior ectopic expression of Shh

If a single nucleotide substitution gives rise to a new *cis*-motif in MFCS1 (Figure S3A in File S1), we inferred that removal of the mutant nucleotide must abolish ectopic *Shh* expression. Alternatively, if the nucleotide substitution causes a degenerative change—in other words, if it is a loss-of-binding type mutation (Figure S3B in File S1)—replacement with any nucleotide or even deletion of the mutant nucleotide might induce ectopic *Shh* expression. Therefore, to further classify the four mouse mutations, we deleted nucleotides at each mutation site in the entire mouse 1.2 kb MFCS1 sequence (Figure 2A). The intact MFCS1 directed *LacZ* expression in the posterior limb bud as previously reported (Figure 2B; Lettice *et al.* 2003). When the nucleotide “T” at the *Hx* mutation was deleted from the *Hx* MFCS1, we observed no ectopic *LacZ* expression in the transgenic mouse (Figure 2C), with normal expression retained in the posterior limb bud. This suggests that a substitution toward the specific nucleotide is required for the *Hx* mutation.

Unlike the *Hx* mutation, transgenic mouse embryos with a single nucleotide deletion at the *M101116*, *M100081*, and *DZ* mutation sites showed anterior *LacZ* expression (Figure 2, D–F and Table S3 in File S2). Thus, a degenerative change including substitution and deletion at these mutation sites retains anterior expression of *Shh*. Notably, in the case of *M101116*, both anterior and posterior expression domains of *LacZ* were markedly expanded, contrasting with the small anterior expression domain in the transgenic embryo with a MFCS1 reporter construct containing a single nucleotide substitution at the original *M101116* mutation (Masuya *et al.* 2007). Removal and substitution of the single nucleotide at the *M101116* site may differently influence the MFCS1 regulatory activity.

The single nucleotide deletion at the *M100081* and *DZ* mutations generated a somewhat weaker anterior expression of *LacZ*, and the ectopic anterior expression was observed less frequently than with the *M101116* mutation (Figure 2, E and F and Table S3 in File S2). The *DZ* mutation is known to newly elicit binding of a nuclear factor, HNRNPU, suggesting that *DZ* is a gain-of-function mutation (Zhao *et al.* 2009). Unexpectedly, in our transgenic assay, deletion of the nucleotide at the *DZ* mutation induced a weak and less frequent anterior ectopic expression of *LacZ* in the limb bud. Because the *DZ* nucleotide is next to the *M100081* nucleotide (Figure S1 in File S1), it is possible that the *DZ* deletion affects binding of a TF to the *M100081* mutation site. Taken together, the PPD phenotypes of *M100081* and *M101116* most likely arise from the loss of repressor-binding at their mutation sites. Given the different patterns of *LacZ* expression by deletions at the *M100081* and *M101116* mutations, the repression of *Shh* must be controlled independently via local sequences of MFCS1.

### Identification of a factor binding to the Hx mutation site

**Figure 3:**
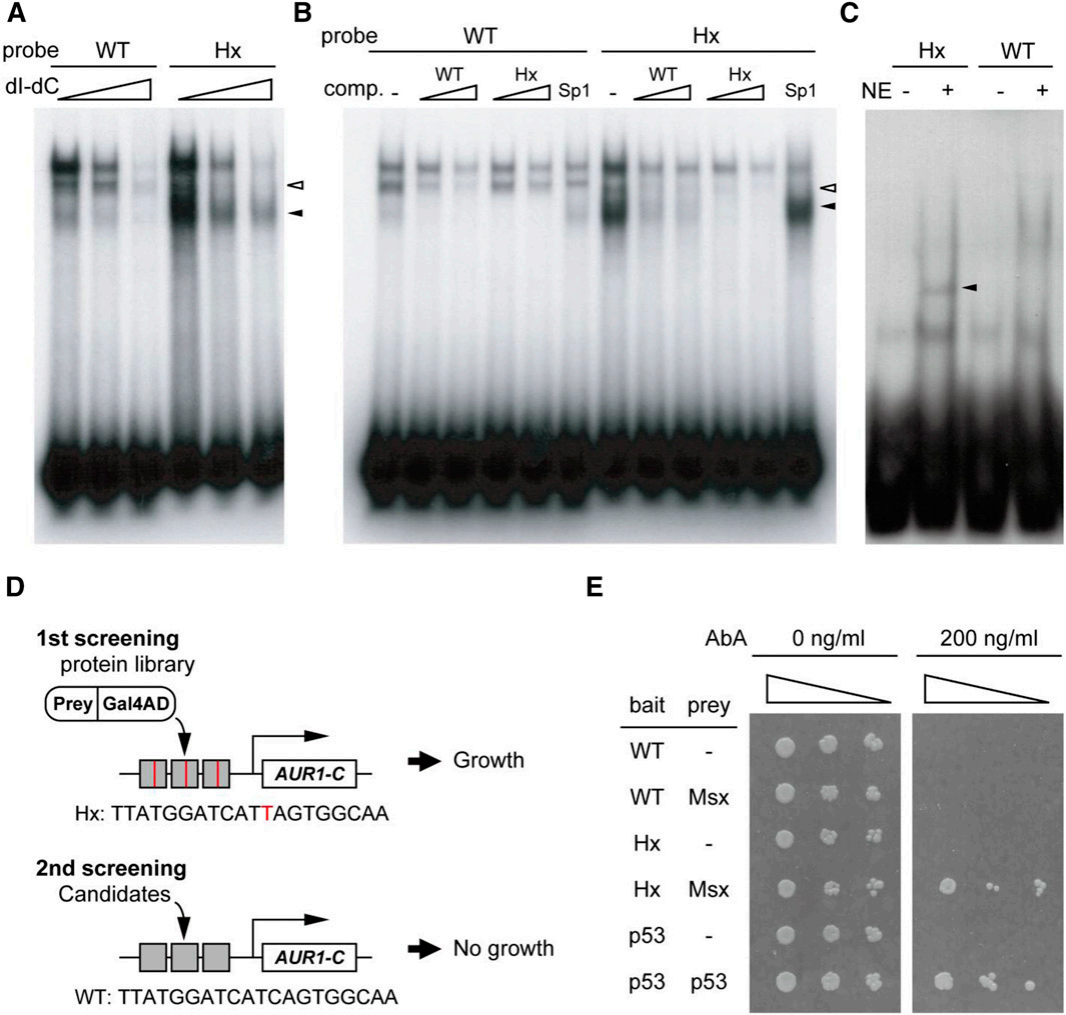
Gain of binding at the *Hx* mutation site. (A–C) EMSA with the sequence of the *Hx* mutation site. Oligo probes were incubated with nuclear extract derived from whole mouse embryos under different concentrations of poly(dI-dC) (A) and cold oligos as competitors [(B) comp.]. Sp1 indicates an SP1-binding fragment used as a control. (C) EMSA with nuclear extract (NE) prepared from E11.5 mouse limb buds. Black arrowheads indicate a specific band shift with the *Hx* probe. (D) A diagram depicting the Y1H assay in this study. In the first screening, three tandem copies of the *Hx* fragment (gray boxes with a red line) were used as bait. To remove pseudopositive clones, a second screening was performed. Surviving clones from the first screening were transformed into the yeast strain containing three tandem copies of the WT fragment (gray boxes). GAL4AD is a GAL4 activation domain. *AUR1-C* is a resistant gene for the selection marker. (E) The *Msx1*-AD clone was transformed into yeast cells containing *Hx*- and WT-bait sequences. Transformants were spotted in serial dilutions (1:10, 1:100, 1:1000) on SD medium lacking leucine (SD/-Leu) under selective (200 ng/ml AbA) or nonselective (-AbA) conditions. Binding of p53 to the p53 motif was used as positive control.

Our transgenic assays suggested that an unknown factor binds specifically to the *Hx* mutation. To assess this, electrophoretic mobility shift assay (EMSA) analysis was performed using nuclear extracts from whole mouse embryos. Both the *Hx* and the WT probes demonstrated a specific band shift at different sizes (Figure 3A, white and black arrowheads). The band shift of the *Hx* probe remained even under the highest concentration of poly(dI-dC), which is a general competitor for nonspecific DNA-binding proteins. This interaction was markedly prevented by adding the cold *Hx*-oligo as a specific competitor (Figure 3B, black arrowhead). This *Hx* mutation-specific band shift was observed in the presence of nuclear extracts from mouse limb buds (Figure 3C). This result suggests that a factor expressed in the limb bud specifically binds to the *Hx* probe, consistent with the transgenic reporter assay. The binding of nuclear factors derived from whole embryos to the WT probe may not be relevant to the limb specific expression of *Shh*.

**Figure 4:**
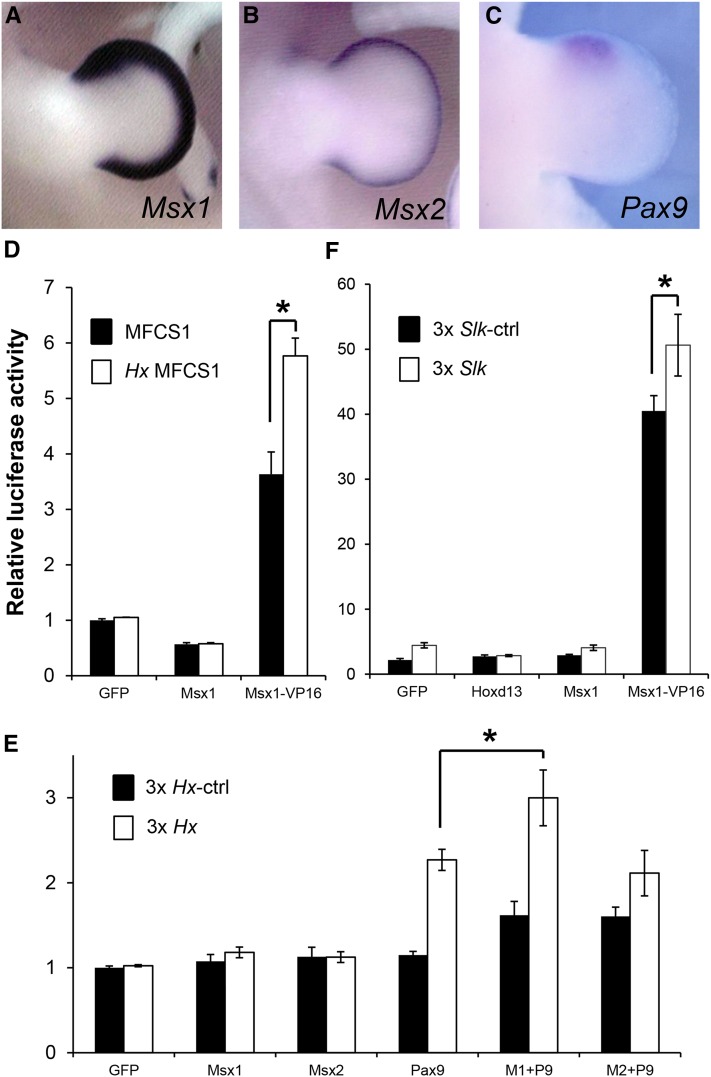
MSX1 binds to the *Hx* mutation site. *In situ* hybridization of E11.5 mouse limb buds with RNA probes for *Msx1* (A) *Msx2* (B) and *Pax9* (C). (D) Relative luciferase activity of the entire MFCS1 (black bars) and *Hx*-MFCS1 (open bars) in NIH3T3 cells. The reference values for cotransfection with *Gfp*-expressing and MFCS1-luc reporter plasmids were set as 1. Error bars represent the SD obtained from three independent experiments. Asterisks show significant differences, as evaluated by Student’s *t*-test (*P* < 0.05). (E) Relative luciferase activity of three tandem repeats of ctrl (black bars) or *Hx* (open bars) fragments in Caco-2 cells. Constructs expressing *Gfp*, *Msx1*, *Msx2*, and *Pax9* were cotransfected with the ctrl- or *Hx*-luciferase constructs. M1, M2, and P9 indicate *Msx1*, *Msx2*, and *Pax9*, respectively. (F) Relative luciferase activity of the three tandem repeats of the 40 bp WT control fragment or the fragment containing the *Slk* mutation in NIH3T3 cells. Constructs expressing *Gfp*, *Hoxd13*, *Msx1*, and the *Msx1–VP16* fusion protein were cotransfected.

To identify this TF, we conducted a yeast one-hybrid assay (Figure 3D). Total RNA was obtained from E11.5 mouse limb buds to construct a plasmid library encoding fusion proteins of limb bud-specific factors and the GAL4 activation domain (AD). After screening, we obtained 15 clones that encode known protein-coding sequences, and the remainder had sequences of intergenic regions or 3′UTRs of genes. To eliminate false positives from the 15 protein-coding clones, we examined the DNA–protein interactions using the WT sequence (Figure 3D). Only one clone was a genuine positive and encoded a fusion protein of the MSX1–GAL4 AD. The E11.5 mouse limb bud expresses two *Msx* family genes, *Msx1* and *Msx2*, (Figure 4, A and B; Davidson *et al.* 1991; Catron *et al.* 1996), which encode homeodomain proteins, consistent with the finding that an ATTA motif is generated by the *Hx* mutation. To further confirm the result of yeast one-hybrid screening, the *Msx1-AD* clone was retransformed into the yeast strains with three tandem repeats of *Hx* and control WT fragments. The yeast cells that have *Msx1-AD* clone and the *Hx* fragment as bait specifically survived under AbA selection (Figure 3E). In general, the MSX1 protein is known as a transcriptional repressor (Catron *et al.* 1995; Lee *et al.* 2004; Wang *et al.* 2011), whereas there are some lines of evidence that MSX1 also activates genes in a context-dependent manner (Andersson *et al.* 2006; Ogawa *et al.* 2006; Zhuang *et al.* 2009). To test the effect of MSX1 on the *Hx* mutation in mammalian cells, we conducted a luciferase reporter assay with 1.2 kb of MFCS1 sequence. Cotransfection of an *Msx1* expression construct with the MFCS1 luciferase or *Hx*-MFCS1-luciferase reporter constructs showed no specific activation of luciferase, as compared with transfection of a *Gfp*-expressing control plasmid (Figure 4D). In contrast, the *Msx1–VP16* construct, which is a fusion protein of MSX1 and a VP16 activation domain, enhanced the reporter activities of both WT- and *Hx*-MFCS1 reporter plasmids (Figure 4D). The significantly higher luciferase activity of the *Hx*-MFCS1 construct compared with that of the WT-MFCS1 construct suggests that MSX1–VP16 binds to the *Hx* mutation site in MFCS1.

*Msx1* is known to activate genes in cooperation with *Pax9* in tooth buds, and with *Pax7* in the cranial neural crest (Ogawa *et al.* 2006; Barembaum and Bronner 2013). In the limb bud, *Pax9* is expressed exclusively in an anterior portion of the mesenchyme (McGlinn *et al.* 2005) where the *LacZ* signal was observed in mouse embryos with the 6× *Hx*-LacZ transgene (Figure 1B and Figure 4C). To test the combined effects of MSX1 and PAX9, *Msx1*- and *Pax9*-expression constructs were cotransfected with the reporter construct containing three tandem repeats of the *Hx* fragment in Caco-2 cells. Either of the *Msx1*- or *Msx2*-expressing constructs alone failed to upregulate luciferase activity, whereas overexpression of *Pax9* resulted in upregulation of the reporter in an *Hx* mutation–dependent fashion. Among the PAX protein family, PAX9 has no homeodomain and its consensus binding motif is quite different from the *Hx* fragment (Jolma *et al.* 2013). Therefore, the upregulation of luciferase by PAX9 may require an additional scaffold protein that binds to an ATTA motif. Moreover, coexpression of *Pax9* with *Msx1* showed significantly higher activation of *Hx*-luc reporter expression than with *Msx2* (Figure 4E). Consistent with a previous study (Ogawa *et al.* 2006), a synergistic action of MSX1 and PAX9 was observed on the *Hx* mutation, at least in cell culture.

Mice with *Msx1* and *Msx2* double-mutations show anterior *Shh* expression in the limb bud (Lallemand *et al.* 2005; Bensoussan-Trigano *et al.* 2011). Therefore, *trans*-acting MSX1 and MSX2 proteins may play a negative role in *Shh* expression during normal limb development. Inversely, our result showed that MSX1 bound to the *Hx* mutation site upregulates target gene expression. The *Hx* mutation might allow interplay between MSX1 and anteriorly expressed PAX9, which does not occur on the MFCS1 enhancer in normal limb development.

### Different influence of MFCS1 mutations on the endogenous Shh regulatory machinery

Clustering of binding motifs for the same TF is a common feature in enhancer sequences (Gotea *et al.* 2010). MFCS1 has multiple binding motifs for the ETS family of TFs. Balanced occupancy between ETS1 and ETV4/5 in MFCS1 might control the expression level of *Shh* in the limb bud. A human PPD mutation in an Australian family (AUS) converts one of the ETV4/5 binding sites into an additional ETS1 binding site, and thereby confers not only an anterior ectopic domain of *Shh*, but also a posterior expansion of the ZPA (Lettice *et al.* 2012). Interestingly, the anterior ectopic domain of *Shh* in the leg bud of the *Slk* mutant is induced secondarily by anterior expansion of the posterior *Shh* in the ZPA (Dunn *et al.* 2011; Johnson *et al.* 2014). The PPD in the human AUS and chicken *Slk* is likely caused by a defect in the mechanism by which the endogenous *Shh* is induced and maintained in the ZPA. In our reporter assay in cultured cells, HOXD13, which is a homeobox protein expressed in the posterior limb bud and an upstream factor for *Shh*, did not activate the luciferase reporter flanked with three tandem repeats of the *Slk* fragment (*Slk*-luc) containing an ATTA motif (Figure 4F). The MSX1–VP16 fusion protein showed *Slk*-dependent activation of the luciferase (Figure 4F), suggesting that MSX1 can bind to the *Slk* mutation site. Posteriorly expressed unknown coactivator(s) that possibly form an activator complex with MSX1 might contribute to PPD in the *Slk* mutant.

In contrast to the AUS and *Slk* mutations, upregulation mediated by the *Hx* mutation is independent of an endogenous mechanism for *Shh* activation in the ZPA. Because the short *Hx* fragment drives no reporter expression in the posterior limb bud, the anterior expansion of posterior *Shh* cannot be the cause of PPD in the *Hx* mutant. This context-independent *Shh* activation in the *Hx* mutation was also observed in the transgenic mouse carrying an inversion of a short segment containing the *Hx* mutation (Lettice *et al.* 2014). *Shh* expression in the ZPA extends distally after the appearance of anterior ectopic *Shh* even in the *Hx* embryo (Blanc *et al.* 2002). This suggests that the anterior ectopic ZPA might reversely affect the posterior *Shh* expression domain, in contrast to that seen in the *Slk* mutant.

In the MFCS1 mouse mutations we studied, *M100081*, *M101116*, and *DZ* involve loss-of-repression (Figure S3B in File S1), and *Hx* involves gain-of-activation (Figure S3A in File S1). Fuxman Bass *et al.* (2015) examined nine human mutations in MFCS1, all of which cause digit malformation, by a high-throughput yeast one-hybrid assay named eY1H. They found that loss or gain of interaction occurs at each human mutation site, as seen in our results for mouse mutations. For instance, the Dutch mutation creates a potential AP2 binding site. Their luciferase assay confirmed that TFAP2B binds to the mutation site, and thereby activates the reporter in cultured cells. However, in this study, the short fragment containing the Dutch mutation did not activate anterior expression in the mouse limb bud (Figure S1D in File S1). The 40 bp fragment is so short that it may lose a necessary element for limb-specific expression. Thus, it is still difficult to examine how each MFCS1 mutation affects regulatory activity of the limb enhancer *in vivo*, although the short fragment containing the *Hx* mutation functions in the mouse embryo.

## Supplementary Material

Supplemental material is available online at www.g3journal.org/lookup/suppl/doi:10.1534/g3.117.044669/-/DC1.
