# Supplementary material for: Two Types of Etiological Mutation in the Limb-Specific Enhancer of Shh
Source: G3 (Bethesda). 2017 Jul 14;7(9):2991–8. doi: 10.1534/g3.117.044669 (PMC5592926; doi:10.1534/g3.117.044669)
Supplement: Supplementary file 1 [file 2991FileS1.pptx]

## Slide 1
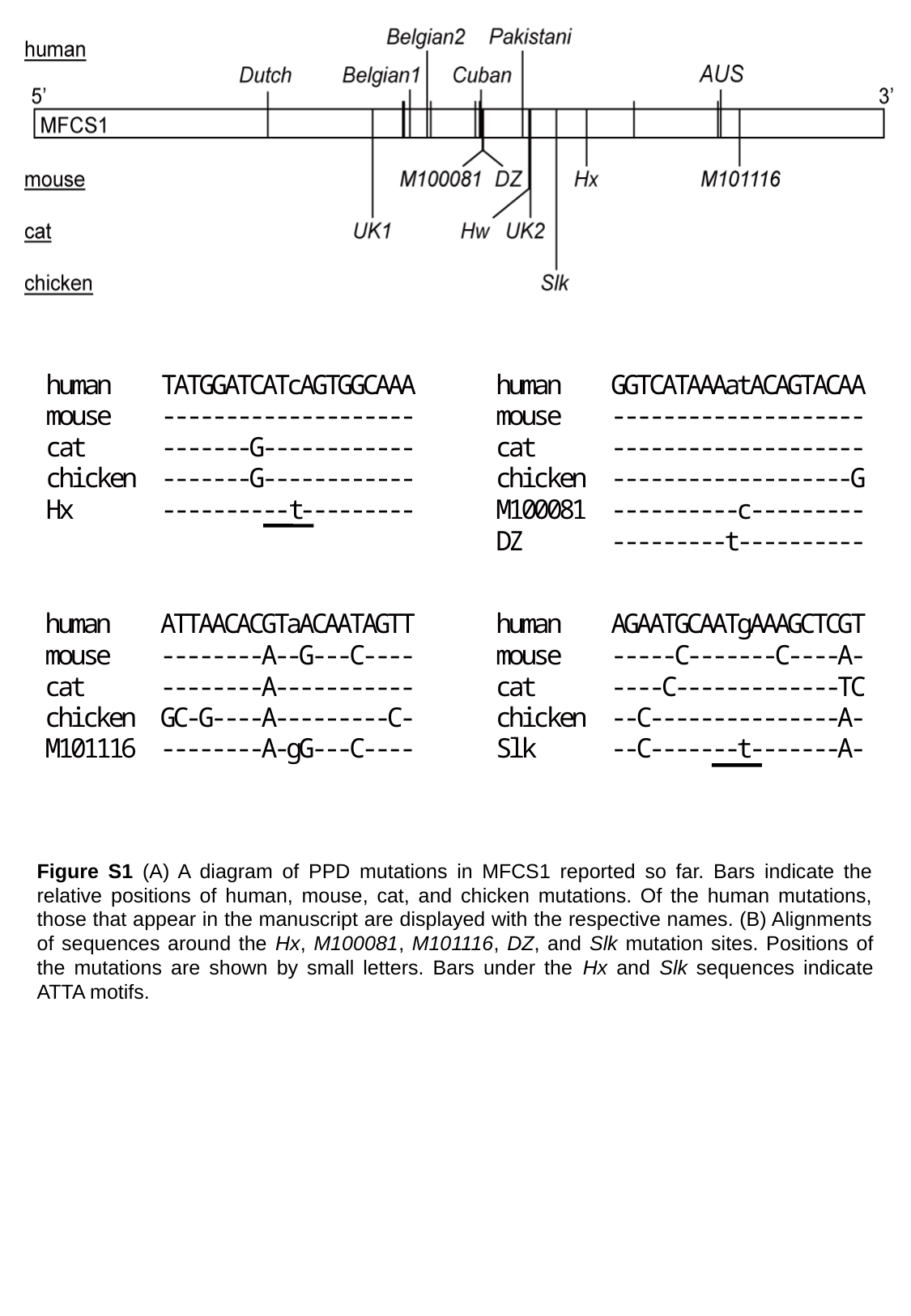

h
u
m
a
n
T
A
T
G
G
A
T
C
A
T
c
A
G
T
G
G
C
A
A
A
h
u
m
a
n
G
G
T
C
A
T
A
A
A
a
t
A
C
A
G
T
A
C
A
A
m
o
u
s
e
-
-
-
-
-
-
-
-
-
-
-
-
-
-
-
-
-
-
-
-
m
o
u
s
e
-
-
-
-
-
-
-
-
-
-
-
-
-
-
-
-
-
-
-
-
c
a
t
-
-
-
-
-
-
-
G
-
-
-
-
-
-
-
-
-
-
-
-
c
a
t
-
-
-
-
-
-
-
-
-
-
-
-
-
-
-
-
-
-
-
-
c
h
i
c
k
e
n
-
-
-
-
-
-
-
G
-
-
-
-
-
-
-
-
-
-
-
-
c
h
i
c
k
e
n
-
-
-
-
-
-
-
-
-
-
-
-
-
-
-
-
-
-
-
G
t
H
x
-
-
-
-
-
-
-
-
-
-
-
-
-
-
-
-
-
-
-
M
1
0
0
0
8
1
-
-
-
-
-
-
-
-
-
-
c
-
-
-
-
-
-
-
-
-
D
Z
-
-
-
-
-
-
-
-
-
t
-
-
-
-
-
-
-
-
-
-
h
u
m
a
n
A
T
T
A
A
C
A
C
G
T
a
A
C
A
A
T
A
G
T
T
h
u
m
a
n
A
G
A
A
T
G
C
A
A
T
g
A
A
A
G
C
T
C
G
T
m
o
u
s
e
-
-
-
-
-
C
-
-
-
-
-
-
-
C
-
-
-
-
A
-
m
o
u
s
e
-
-
-
-
-
-
-
-
A
-
-
G
-
-
-
C
-
-
-
-
c
a
t
-
-
-
-
-
-
-
-
A
-
-
-
-
-
-
-
-
-
-
-
c
a
t
-
-
-
-
C
-
-
-
-
-
-
-
-
-
-
-
-
-
T
C
c
h
i
c
k
e
n
G
C
-
G
-
-
-
-
A
-
-
-
-
-
-
-
-
-
C
-
c
h
i
c
k
e
n
-
-
C
-
-
-
-
-
-
-
-
-
-
-
-
-
-
-
A
-
M
1
0
1
1
1
6
-
-
-
-
-
-
-
-
A
-
g
G
-
-
-
C
-
-
-
-
S
l
k
-
-
C
-
-
-
-
-
-
-
t
-
-
-
-
-
-
-
A
-
Figure S1 (A) A diagram of PPD mutations in MFCS1 reported so far. Bars indicate the relative positions of human, mouse, cat, and chicken mutations. Of the human mutations, those that appear in the manuscript are displayed with the respective names. (B) Alignments of sequences around the Hx, M100081, M101116, DZ, and Slk mutation sites. Positions of the mutations are shown by small letters. Bars under the Hx and Slk sequences indicate ATTA motifs.

## Slide 2
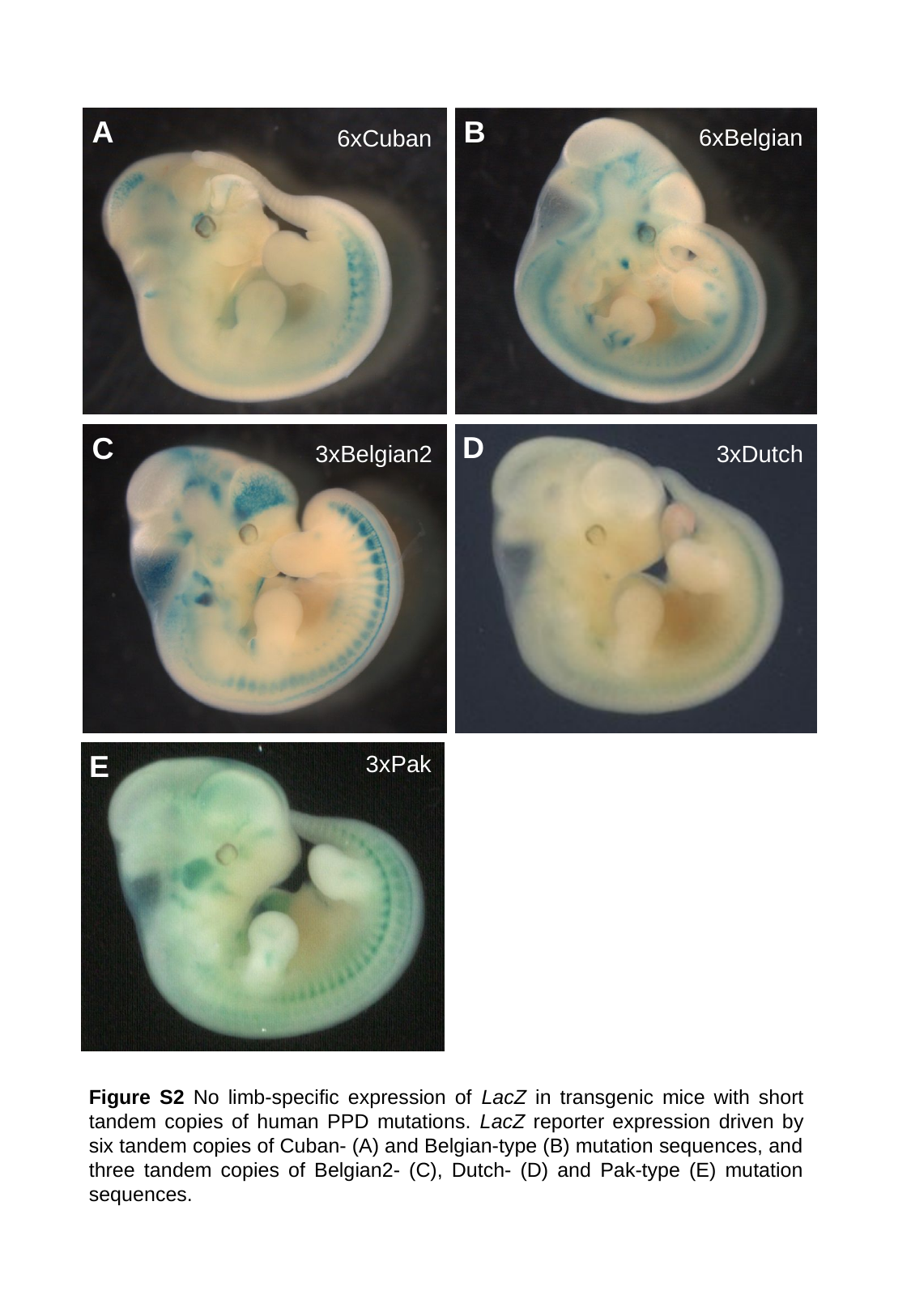

A
B
6xBelgian
6xCuban
D
C
3xBelgian2
3xDutch
E
3xPak
Figure S2 No limb-specific expression of LacZ in transgenic mice with short tandem copies of human PPD mutations. LacZ reporter expression driven by six tandem copies of Cuban- (A) and Belgian-type (B) mutation sequences, and three tandem copies of Belgian2- (C), Dutch- (D) and Pak-type (E) mutation sequences.

## Slide 3
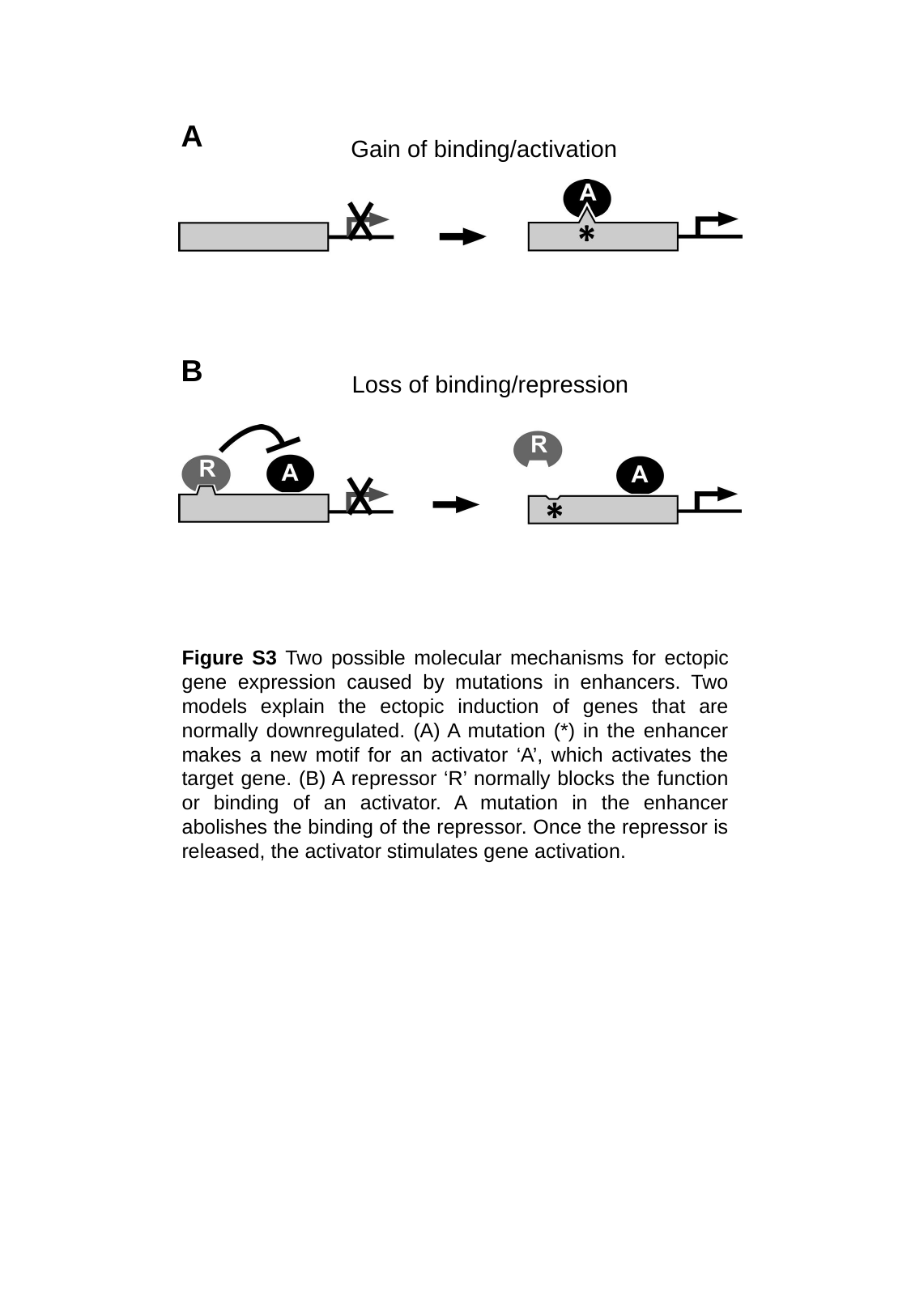

A
Gain of binding/activation
B
Loss of binding/repression
Figure S3 Two possible molecular mechanisms for ectopic gene expression caused by mutations in enhancers. Two models explain the ectopic induction of genes that are normally downregulated. (A) A mutation (*) in the enhancer makes a new motif for an activator ‘A’, which activates the target gene. (B) A repressor ‘R’ normally blocks the function or binding of an activator. A mutation in the enhancer abolishes the binding of the repressor. Once the repressor is released, the activator stimulates gene activation.
