# Supplementary material for: Two Types of Etiological Mutation in the Limb-Specific Enhancer of Shh
Source: G3 (Bethesda). 2017 Jul 14;7(9):2991–8. doi: 10.1534/g3.117.044669 (PMC5592926; doi:10.1534/g3.117.044669)
Supplement: Supplementary file 2 [file 2991FileS2.docx]

**Table S1 MFCS1 fragments used for reporter Tg assay**

|  | **sequence (5’ – 3’)** |
| --- | --- |
| Hx | TTTCCAACAATTTATGGATCATTAGTGGCAAAAAAAACAA |
| Hx-ctrl | TTTCCAACAATTTATGGATCATCAGTGGCAAAAAAAACAA |
| M100081 | AGTCATCTGGTCATAAAACACAGTACAAGGTCACTTTTAT |
| 81-ctrl | AGTCATCTGGTCATAAAATACAGTACAAGGTCACTTTTAT |
| M101116 | GAGAATCAAATTAACACATGGCAACAGTTAGTGAGATATG |
| 1116-ctrl | GAGAATCAAATTAACACATAGCAACAGTTAGTGAGATATG |
| Cuban | AGTCATCTGGTCATAAAATATAGTACAAGGTCACTTTTAT |
| Belgian | CATATTAAAACGATCTTAGATCCTCCTCTATTGTGCTGTC |
| Belgian2 | CTCAAACTGTGACACAGGATGGAAACATATTAAAACGATC |
| Dutch | ATCCTATAGATCATGTGTGAGCTTCTGGACACTCTAGGAT |
| Pak | ACTCACTCTGTGTCTCTTTACGATGGAGGCCTGATACAAA |
| Slk | GGTTTGTCTCAATGAGCTTTAATTGCATGCTTTCATTATT |
| Slk-ctrl | GGTTTGTCTCAATGAGCTTTCATTGCATGCTTTCATTATT |

**Table S2 Reporter expression in tandem copy Tg mice**

|  | **anterior**  **edge** | **ZPA** | **total Tg** |
| --- | --- | --- | --- |
| Hx | 15 | 0 | 17 |
| Hx-ctrl | 0 | 0 | 4 |
| M100081 | 0 | 0 | 8 |
| 81-ctrl | 0 | 2 | 7 |
| M101116 | 0 | 0 | 8 |
| 1116-ctrl | 0 | 0 | 9 |
| Cuban | 0 | 0 | 7 |
| Belgian | 0 | 0 | 7 |
| Belgian2 | 0 | 0 | 8 |
| Dutch | 0 | 0 | 4 |
| Pak | 0 | 0 | 6 |
| Slk | 0 | 0 | 3 |

**Table S3 Reporter expression in Tg mice with a point deletion**

|  | **anterior edge** | **ZPA** | **total Tg** |
| --- | --- | --- | --- |
| WT MFCS1 | 0 | 6 | 6 |
| Hx | 0 | 12 | 12 |
| M100081 | 3 | 8 | 8 |
| M101116 | 10 | 10 | 10 |
| DZ | 2 | 7 | 7 |
